# Interactive Multiobjective Procedure for Mixed Problems and Its Application to Capacity Planning

**DOI:** 10.3390/e23101243

**Published:** 2021-09-24

**Authors:** Maciej Nowak, Tadeusz Trzaskalik, Sebastian Sitarz

**Affiliations:** 1 Department of Operations Research, University of Economics in Katowice, Ul. 1 Maja 50, 40-287 Katowice, Poland; tadeusz.trzaskalik@ue.katowice.pl; 2 Independent Researcher, 41-406 Mysłowice, Poland; sitarzseba@gmail.com

**Keywords:** ordered structures, multiobjective dynamic programming, scalarization, interactive procedure, trade-offs, capacity planning

## Abstract

A problem that appears in many decision models is that of the simultaneous occurrence of deterministic, stochastic, and fuzzy values in the set of multidimensional evaluations. Such problems will be called mixed problems. They lead to the formulation of optimization problems in ordered structures and their scalarization. The aim of the paper is to present an interactive procedure with trade-offs for mixed problems, which helps the decision-maker to make a final decision. Its basic advantage consists of simplicity: after having obtained the solution proposed, the decision-maker should determine whether it is satisfactory and if not, how it should be improved by indicating the criteria whose values should be improved, the criteria whose values cannot be made worse, and the criteria whose values can be made worse. The procedure is applied in solving capacity planning treated as a mixed dynamic programming problem.

## 1. Introduction

Many decision problems are formulated as multiobjective optimization problems. Their goal is to find the set of non-dominated solutions in the criteria space and the corresponding set of efficient solutions in the decision space, as well as to assist the decision-maker in the selection of the final decision.

A problem that appears in many multiobjective optimization models is that of the simultaneous occurrence of different types of data. We will call them mixed problems. Examples of such situations are described in [1]. We can encounter them in a conceptual framework for the evaluation of health risks. The qualitative risk assessment is based on experts’ knowledge and is rather fuzzy in contrast to quantitative risk, the assessment of which is probabilistic. Another example is the military problem of choosing the best course of action to stop aerospace violations. The commander has to consider several attributes of a different nature: deterministic (cost of equipment), probabilistic (risk of loss of pilots in battle or equipment break-down), or fuzzy (risk of bad timing in resource deployment). In the current paper, we will take care of the capacity-planning problem. We will consider deterministic (total value of investment expenditure), stochastic (sum of discounted cash flow, the mean level of customer demand fulfillment, or mean level of production capacity usage), and fuzzy criteria (total labor cost related to the preparation of the investment process). Approaches applied for solving mixed problems can be found in [1,2,3,4]. Mixed problems lead to multiobjective optimization problems formulated in ordered structures. Different approaches based on various types of orders are presented in [5,6,7,8,9,10].

An essential issue here is the scalarization problem, which appears, in various guises, in the literature dealing with multicriteria decision-aiding.

In the case of real vectors, scalarization was considered in goal programming problems [11], the ε-constraint method [12], minimizing the distance using an ideal solution [13], the lexicographic method [14], and evolutionary algorithms for multiobjective optimization [15]. A review of scalarization methods on multiobjective optimization based on weights, presenting three types of weights: equal weights, rank order centroid weights, and rank-sum weights, was given in [16].

In the case of fuzzy data, scalarization was considered in the multiobjective programming problems with fuzzy coefficients using the embedding theorem and the concept of convex cone [17], as well as in the ε-constraint method and weighted sum method in fuzzy multiobjective programming [18]. Kon [19] studied connections between set optimization problems and scalarization of fuzzy optimization problems.

In the case of stochastic data, some models of scalarization in stochastic multiobjective programming problems were examined by Caballero et al. [20]. Scalarization based on expected value, variance, and Tammer Optimality was proposed by Adeyefa and Luhandjula [21]. Noyan and Rudolf [22] proposed a new class of scalarization functions in optimization with stochastic preferences based on coherent risk measures and second-order stochastic dominance. Scalarization approaches in stochastic multiobjective problems were considered by Kankova [23,24].

Finding all non-dominated solutions is very often insufficient for the decision-maker, who has to select the final decision. For that reason, various suggestions appear, assisting the DM in this matter.

One of the frequently used methods of solving multiobjective problems is the interactive approach, which was being developed starting with the STEM method of Benayoun et al. [25], through the methods proposed in [26,27,28,29].

The term “trade-off” is widely used in decision-making. It usually refers to a conflict between two criteria [30,31,32]. The notion “value trade-offs” is used by Keeney [33] to express how much the DM is inclined to decrease one criterion to achieve a particular increase in another criterion. When this approach is used, the DM articulates his/her preferences by specifying trade-off values he/she considers satisfactory [34]. The term “trading-off” is also used to illustrate the situation of a DM who agrees to reduce the value of one criterion in order to improve the value of another [35]. In this study, the term “trade-off” is defined as a ratio determining how much the value of one criterion is increased per a unit of decrease in the value of another criterion when a particular solution is replaced by another [36,37]. An approach based on the trade-off analysis has also been used in interactive methods [37,38].

In the present paper, we will deal with the possibility of applying an interactive approach, based on trade-offs, to the solution of mixed problems. This requires, first, to determine the set of efficient solutions to the decision problem in question.

The interactive approach proposed in the paper will be used to solve the problem of capacity planning. Capacity is defined as the maximum level of value-added activity over a period of time that the operation can achieve under normal conditions [39]. For top management, capacity decisions are of primary importance, as they determine whether the organization will be able to meet the demand and how effectively it will use its resources.

Two characteristics of capacity, lead-time and economics of scale, must be taken into account when planning changes in capacity. As increasing capacity takes time, decisions need to be made before demand levels can be estimated precisely. On the other hand, there is pressure to make a change in capacity large enough to exploit economies of scale. Thus, two questions must be answered: when to make a change and how large the capacity increments should be.

The possibility of applying dynamic programming methods for the planning of production capacities was used in the past. Erlenkotter [40] proposed two approaches for dynamic capacity-planning problems with many locations. This was applied to a large-scale problem of planning capacity expansion for India’s nitrogenous fertilizer industry. Herbots et al. [41] investigated capacity planning under limited regular and non-regular resources, with a finite planning horizon taken into consideration. Lin et al. [42] considered the dynamic multi-site capacity planning problem in the thin film transistor liquid crystal display industry under stochastic demand, which follows Markov properties. Wang and Nguyen [43] proposed a stochastic dynamic programming solution to a decision-making problem related to technology replacement policy and a capacity plan of resources to satisfy customer demand under technological changes in conjunction with integer programming for simultaneous capacity planning.

Capacity planning methodology is often applied to waste management. Baetz [44] presented a dynamic programming model for determining the optimal capacity expansion patterns for waste-to-energy and landfill facilities over time. Huang [45] introduced a grey dynamic programming method by incorporating concepts of grey systems and grey decisions within a dynamic programming framework as a means for decision making under uncertainty. Nie et al. [46] generated interval solutions for capacity expansion of waste management facilities and the relevant waste-flow allocation. Dai et al. [47] developed an interval-parameter, chance-constrained dynamic programming method for the capacity planning of an integrated municipal solid waste management system under uncertainty. 

There are also other new relevant approaches in the field worth to be mentioned. For example, in [48], optimal computing budget allocation for the vector-evaluated genetic algorithm in multiobjective simulation optimization is considered. In [49], bankruptcy prediction for small and medium enterprises using transactional data in the multiobjective framework is shown. In [50], three portfolio selection strategies for loss-averse investors: semi-variance, conditional value-at-risk, and a combination of both risk measures are analyzed.

Later in the paper, the capacity-planning problem will be considered as a mixed, multiobjective, multistage decision process. This problem leads to the formulation of dynamical optimization problems in ordered structures. The first paper in this field was Brown and Strauch [51], which presented the application of the optimality principle for a class of multicriteria dynamic programming problems with a lattice order. Mitten [52] showed a method for solving multistage decision process in which the real-valued objective function was replaced by a preference relation. Cangpu [53] studied the dynamic property of the efficient solutions of dynamic systems and showed that they possessed a chain property necessary for establishing the fundamental equation. Henig [54] defined a dynamic model with returns in a partially ordered set and showed that Bellman’s principle of optimality is valid with respect to maximal returns. Assumptions of separability and monotonicity of MCDM were needed to guarantee that each non-dominated solution could be computed. Trzaskalik and Sitarz [55,56] dealt with the solution of the problem of searching for optimal solutions in ordered spaces. They presented optimality equations and examples of ordered mixed structures, including structures with simultaneous deterministic, stochastic, and fuzzy criteria. Optimality based on lattice theory in dynamic programming is also shown in [57].

Despite the fact that the application of Bellman’s optimality equations allows generating a complete set of efficient solutions of a multiobjective dynamic programming problem, this set is usually too numerous to be useful for the decision-maker (DM) in the making of the final decision, and for that reason, it is necessary to narrow it down. To achieve this goal, we will use the interactive procedure proposed in this paper.

The aim of the paper is to present an interactive procedure with trade-offs that help the DM to make a final decision. The procedure is applied to solving the capacity planning problem. The description of the interactive procedure that can be applied to any mixed problem and the example of its application to capacity-planning problems are the main contribution of the paper.

The paper consists of six sections. Section 1 is an introduction to the problems discussed. In Section 2, we provide a definition of efficient decisions in ordered structures. Section 3 describes an interactive procedure based on the application of trade-offs, which allows obtaining the final solution interactively with the DM. In Section 4, capacity planning is formulated as a mixed multiobjective dynamic programming problem, and a numerical example is formulated. In Section 5, an application of the proposed interactive method is shown. Conclusions in Section 6 end the paper.

## 2. Ordered Structures and Scalarization

### 2.1. Ordered Structures

We introduce the following notation. Let *D* be a decision space, which consists of the finite number of solutions (decisions). We consider a multi-criteria problem and a set of *K* criteria: $\left\{\alpha^{1},\alpha^{2},\ldots ,\alpha^{K}\right\}\;$:

(1) $$\alpha^{k}:D\to W^{k}\;\mathrm{for}\;k\in \bar{1,\;K}.$$

We denote the set of integers {1,…,K} as $\bar{1,\;K}$.

We focus on structures consisting of sets $W^{k}$, operators $\circ^{k}$, and relations $\leq^{k}$. We assume that these structures satisfy the following conditions: $\left(W^{k},\leq^{k},\circ^{k}\right)\;$ is a partially ordered set for $k\in \bar{1,\;K}$; for all elements a,b,c of set *W* and for all $k\in \bar{1,\;K}$ the following holds:

(2) $$\begin{array}{c} a\circ^{k}\left(b\circ^{k}c\right)=\left(a\circ^{k}b\right)\circ^{k}c,\\ a\leq^{k}b\;\Rightarrow \;a\circ^{k}c\leq^{k}b\circ^{k}c\;\wedge \;c\circ^{k}a\leq^{k}c\circ^{k}b. \end{array}$$

Let $\left(W,\leq^{W},\circ^{W}\right)$ be the Cartesian product of structures $\left(W^{k},\leq^{k},\circ^{k}\right)$, i.e.,

(3) $$\left(W,\leq^{W},\circ^{W}\right)=\left(W^{1},\leq^{1},\circ^{1}\right)\times \left(W^{2},\leq^{2},\circ^{2}\right)\times \ldots \times \left(W^{K},\leq^{K},\circ^{K}\right).$$

Furthermore, $\left(W,\leq^{W},\circ^{W}\right)$ is an ordered structure, being the Cartesian product of ordered structures [58]. Several examples of ordered structures are given by Trzaskalik and Sitarz in [56]. Below, we present an example of an ordered structure consisting of mixed data: stochastic, fuzzy, and real.

**Example** **1.** *We consider the following structure*:

(4) $$W=W^{1}\times W^{2}\times W^{3}\times W^{4}\times W^{5}$$

*where*$W^{1},W^{2}$*and*$W^{3}$*are the sets of random variables with finite sets of realizations,*$W^{4}$* is the set of all real numbers, and *$W^{5}$* is the set of all triangular fuzzy numbers. As the operators in these sets, we take the sum of random variables, denoted by “*${+}_{RV}$*”, the sum of real numbers, denoted by “*+*”, and the sum of triangular fuzzy numbers, denoted by “*${+}_{TFN}$*”. Thus, the operator in the set W has the following form:*

(5) $$\circ^{W}=\left({+}_{\mathrm{RV}}\right)\times \left({+}_{\mathrm{RV}}\right)\times \left({+}_{\mathrm{RV}}\right)\times \left(+\right)\times \left({+}_{\mathrm{TFN}}\right).$$

*We compare random variables by using the first-order stochastic dominance*$\left(\leq_{FSD}\right)$*, see for example,* [59,60]*. The relation in the set of triangular fuzzy numbers is based on the comparison of the parameters of these fuzzy numbers* $\left(\leq_{TFN}\right)$*; for more information, see* [61,62]. *Moreover, we compare real numbers in a classical way* (≤)*. We obtain the following relation in W:*

(6) $$\leq^{W}=\left(\leq_{\mathrm{FSD}}\right)\times \left(\leq_{\mathrm{FSD}}\right)\times \left(\leq_{\mathrm{FSD}}\right)\times \left(\leq \right)\times \left(\leq_{\mathrm{TFN}}\right).$$

*The sets*$W^{1},W^{2}$*and*$W^{3}$*with addition and FSD are ordered structures; for details, see* [59]. *The set of real numbers* $W^{4}$ *with the operator and relation defined above is an ordered structure. The set* $W^{5}$ *with addition and the comparison relation described above is an ordered structure; see* [61]. *Thus, we have here an ordered structure with mixed data: stochastic, real, and fuzzy.*

Let $\alpha =\left(\alpha^{1},\alpha^{2},\ldots ,\alpha^{K}\right)$ be a vector function. We consider the set of elements α(D)⊂W. The set of maximal elements of α(D) is defined as follows:

(7) $$\max\;\alpha \left(D\right)=\left\{d^{*}\in D:\sim \exists_{d’\in D}\;\alpha \left(d^{*}\right)\leq \alpha \left(d’\right)\wedge \alpha \left(d^{*}\right)\neq \alpha \left(d’\right)\right\}.$$

Let

(8) $$D^{*}=\mathrm{argmax}\;\alpha \left(D\right).$$

The set $D^{*}$ is the set of all efficient solutions in the decision space. 

### 2.2. Scalarization

Let us consider set $W^{k}$ for $k\in \bar{1,\;K}$ and a real-valued function

(9) $$\beta^{k}:\;W^{k}\to \;ℜ$$

where ℜ is the set of real numbers. Function $\beta^{k}$ is called scalarization operator for criterion $\alpha^{k}$, whereas value $f^{k}\left(d\right)=\beta^{k}\left(\alpha^{k}\left(d\right)\right)$ is called the scalarized value for the solution d and criterion $\alpha^{k}$. Vector

(10) $$f\left(d\right)=\left(f^{1}\left(d\right),f^{2}\left(d\right),\ldots ,f^{K}\left(d\right)\right)\;$$

is the vector of scalarized values for the solution d. The scalarized vector criteria function has the form:

(11) $$f=\left(f^{1},f^{2},\ldots ,f^{K}\right)\;$$

and consists of components $f^{1},f^{2},\ldots ,f^{K}$, which are scalarized criteria. Later on, they will be treated as a set of functions:

(12) $$F=\left\{f^{1},f^{2},\ldots ,f^{K}\right\}.$$

**Example** **2.** *We consider the ordered structure described in Example 1:*

(13) $$W=W^{1}\times W^{2}\times W^{3}\times W^{4}\times W^{5}\;$$

*where* $W^{1},W^{2}$ *and* $W^{3}$ *are the sets of random variables with finite sets of realizations,* $W^{4}$ *is the set of all real numbers, and* $W^{5}$ *is the set of all triangular fuzzy numbers. Applied scalarization operators* $\beta^{k}$ *are as follows. For criteria* $\alpha^{1},\alpha^{2}$ *, and* $\alpha^{3}$ *,* *the assigned scalarization operators* $\beta^{1},\beta^{2}$ *,* *and* $\beta^{3}$ *are expected values of random variables. As the set* $W^{4}$ *is the set of real numbers, for criterion* $\alpha^{4}$ *we assign the scalarization operator* $\beta^{4}$ *as the identity function. The set of triangular fuzzy numbers* $W^{5}$ *consists of triples of real numbers: center and two spreads (left and right). For criterion* $\alpha^{5}$ *,* *as the scalarization operator* $\beta^{5}$ *,* *we will take the center of the map assigned to each fuzzy number. *

*For any solution* d *, the vector of scalarized values can be written as follows:*

(14) $$\begin{array}{c} f\left(d\right)=\left(f^{1}\left(d\right),f^{4}\left(d\right),f^{3}\left(d\right),f^{4}\left(d\right),f^{5}\left(d\right)\right)=\\ \left(\beta^{1}\left(\alpha^{1}\left(d\right)\right),\beta^{2}\left(\alpha^{2}\left(d\right)\right),\beta^{3}\left(\alpha^{3}\left(d\right)\right),\beta^{4}\left(\alpha^{4}\left(d\right)\right),\beta^{5}\left(\alpha^{5}\left(d\right)\right)\right). \end{array}$$

*The set of scalarized criteria is:*

(15) $$F=\left\{f^{1},f^{2},f^{3},f^{4},f^{5}\right\}.$$

## 3. An Interactive Procedure Based on An Analysis of Trade-Offs

### 3.1. Trade-Offs Analysis

Let $D=\left\{d_{1},d_{2},\ldots ,d_{N}\right\}$ be the set of efficient solutions and *N* the number of efficient solutions. Let $F_{\max}$ be the set of the scalarized criteria for which the largest values are preferred, and $F_{\min}$ be the set of the scalarized criteria for which the smallest values are preferred; we have

(16) $$F=F_{\max}\cup^{\;}F_{\min}.$$

For simplicity, in what follows, we will call the scalarized criteria *f_k_* simply “criteria”.

Our procedure uses trade-offs to identify the solutions proposed to the DM. The trade-off $t_{kl}\left(d_{i},d_{j}\right)$ is calculated for the pair of solutions $\left(d_{i},d_{j}\right)$ and a pair of criteria $\left(f^{k},f^{l}\right)$, such that $d_{i}$ is evaluated as better than $d_{j}$ with respect to $f^{k}$, but worse with respect to $f^{l}$. It determines the value by which $f^{k}$ will change per unit of change of $f^{l}$ when $d_{j}$ is replaced by $d_{i}$.

The application of trade-offs in a two-criteria problem is fairly obvious. Let us assume that solution $d_{j}$ has been selected and presented to the DM, with the information that a simultaneous improvement of both criteria is not possible. Once the DM knows the value of both criteria, he/she states that $f^{k}$ should be improved at the expense of $f^{l}$. If the DM does not formulate his/her expectations in any other way, then as the next proposal for him/her, we can select, from among those solutions for which the value of $f^{k}$ is better than it is for $d_{j}$, the one for which the trade-off is the highest.

If more than two criteria are considered, the application of trade-offs is not obvious. Assume that the DM is given a solution $d_{j}$ and is considering whether it is worth replacing it with another solution $d_{i}$. In the proposed procedure, we assume that in each iteration, the DM evaluates the proposed solution and either accepts it as the final solution or determines how it should be improved. He/she provides the relevant information by dividing the criteria into three groups: those whose values should be improved (the set $F_{1}$), those whose values should be at least preserved at the current level (the set $F_{2}$), and those whose values can be made worse (the set $F_{3}$). Next, all the solutions which satisfy these requirements are determined. If such solutions do not exist, the DM is asked to correct his/her requirements. On the other hand, if there are more than two such solutions, trade-offs are used to determine another candidate solution, calculated for each pair of criteria $\left(f^{k},f^{l}\right)$ such that $f^{k}\in F_{1}$ and $f^{l}\in F_{3}$.

The question arises of how to compare trade-offs calculated for various pairs of criteria. There are two problems here. First, the values of trade-offs calculated for various criteria pairs can be incomparable because of various units used to measure various criteria. This problem is relatively easy to solve since criteria values can be standardized. The second problem is more complex: for some criteria pairs, the calculation of trade-offs can be invalid.

**Example** **3.** *Let us consider the situation presented in Table 1.*

*The DM, presented with solution*$d_{1}$*, stated that the value of*$f^{1}$*should be higher, while the values of the remaining criteria can be lowered. This means that*$F_{1}=\left\{f^{1}\right\}$*,*$F_{2}=\emptyset$*,*$F_{3}=\left\{f^{2},f^{3}\right\}$*. There are three solutions for which the value of the first criterion exceeds the value obtained for*$d_{1}$*:*$d_{2}$*,*$d_{3}$*and*$d_{4}$*. To determine which of them should be proposed next to the DM, the trade-offs should be calculated for each of them for two criteria pairs:*$\left(f^{1},f^{2}\right)$*and*$\left(f^{1},f^{3}\right)$.

*It is easy to notice that while the replacement of*$d_{1}$*by*$d_{4}$*results in decreasing both*$f^{2}$*and*$f^{3}$*, selecting*$d_{2}$*or*$d_{3}$*results in decreasing only one of them. Due to that, it makes no sense to calculate the trade-off for criteria pair*$\left(f^{1},f^{3}\right)$*and solution*$d_{2}$*, and also for the criteria pair*$\left(f^{1},f^{2}\right)$*and solution*$d_{3}$.

In a situation such as outlined above, we propose to calculate trade-offs whenever it is reasonable, e.g., when the increase of one criterion is accompanied by a decrease in the other. Whereas for when a particular solution the values of both criteria increase, the trade-off is taken to be twice the maximum value of the trade-off calculated for the remaining solutions. Finally, for each solution that meets the requirements formulated by the DM, the average of trade-offs computed for various pairs of criteria is calculated, and a solution maximizing the average is taken as a new proposal for the DM. 

During this procedure, the DM is presented with scalarized evaluation values with respect to the individual criteria. Nonetheless, to make it possible to compare trade-offs obtained for various criteria pairs, we will also use standardized criteria values, calculated as follows:

(17) $$g^{k}\left(d_{i}\right)=\{\begin{array}{cc} \frac{f^{k}\left(d_{i}\right)-\underset{j\in \bar{1,N}}{\min}\left\{f^{k}\left(d_{j}\right)\right\}}{\underset{j\in \bar{1,N}}{\max}\left\{f^{k}\left(d_{j}\right)\right\}-\underset{j\in \bar{1,N}}{\min}\left\{f^{k}\left(d_{j}\right)\right\}} & \text{for }f^{k}\in F_{\text{max}}\\ \frac{\underset{j\in \bar{1,N}}{\max}\left\{f^{k}\left(d_{j}\right)\right\}-f^{k}\left(d_{i}\right)}{\underset{j\in \bar{1,N}}{\max}\left\{f^{k}\left(d_{j}\right)\right\}-\underset{j\in \bar{1,N}}{\min}\left\{f^{k}\left(d_{j}\right)\right\}} & \text{for }f^{k}\in F_{\text{min}} \end{array}$$

In order to standardize the values of criteria that are maximized, we divide the difference between the value for a particular solution $d_{i}$ and the minimal value for this criterion by the difference between the maximal and minimal value of this criterion. On the other hand, in order to standardize the values of criteria that are minimized, we divide the difference between the minimum value for this criterion and the value for a particular solution $d_{i}$ by the difference between maximal and minimal value of this criterion. Thus the standardized values of all the criteria belong to the interval [0, 1], with the value 0 assigned to the worst solution, and the value 1 to the best solution with respect to the given criterion.

The trade-off is calculated from the following formula:

(18) $$t_{kl}\left(d_{i},d_{j}\right)=\frac{g^{k}\left(d_{i}\right)-g^{k}\left(d_{j}\right)}{g^{l}\left(d_{j}\right)-g^{l}\left(d_{i}\right)}$$

The trade-off is calculated for standardized values of criteria. It determines the value by which $g^{k}$ will change per unit of change of $g^{l}$ when $d_{j}$ is replaced by $d_{i}$. The greater the trade-off, the greater the increase in $g^{k}$ per unit decrease in $g^{l}$.

Let $D^{\left(q\right)}$ be the set of solutions considered in iteration q. In each iteration, the DM is shown a candidate solution $d^{\left(q\right)}$ and a potency matrix $M^{\left(q\right)}$ with two rows: the first one groups the best values of the criteria for the solutions from set $D^{\left(q\right)}$, and the second one, the worst values:

(19) $$M^{\left(q\right)}=\left[\begin{array}{ccc} {\bar{f}}^{1\left(q\right)} & \cdots & {\bar{f}}^{K\left(q\right)}\\ {\underline{f}}^{1\left(q\right)} & \cdots & {\underline{f}}^{K\left(q\right)} \end{array}\right],$$

where:

(20) $$\begin{array}{c} {\bar{f}}^{k\left(q\right)}=\{\begin{array}{cc} \underset{d_{i}\in D^{\left(q\right)}}{\max}f^{k}\left(d_{i}\right) & \mathrm{for}\;f^{k}\in F_{\max}\\ \underset{d_{i}\in D^{\left(q\right)}}{\min}f^{k}\left(d_{i}\right) & \mathrm{for}\;f^{k}\in F_{\min} \end{array},\\ {\underline{f}}^{k\left(q\right)}=\{\begin{array}{cc} \underset{d_{i}\in D^{\left(q\right)}}{\min}f^{k}\left(d_{i}\right) & \mathrm{for}\;f^{k}\in F_{\max}\\ \underset{d_{i}\in D^{\left(q\right)}}{\max}f^{k}\left(d_{i}\right) & \mathrm{for}\;f^{k}\in F_{\min} \end{array}. \end{array}$$

to return to the solution considered in previous iterations if it is considered better than the current proposal.

### 3.2. Description of the Interactive Procedure

We define set *S* as a set that contains all the solutions proposed to the DM till now. This allows the *DM* to return to the solution considered in previous iterations if it is considered better than the current proposal. At the beginning of the procedure, set *S* is empty. 

The proposed interactive procedure consists of the following steps:

*Preliminary stage*

1. For each efficient solution, calculate $f^{k}\left(d_{i}\right)$, $k\in \bar{1,\;K}$, $i\in \bar{1,\;N}$.
2. Calculate the standardized criteria values $g^{k}\left(d_{i}\right)$, for all the efficient solutions, $k\in \bar{1,\;K}$, $i\in \bar{1,\;N}$.
3. Determine the first candidate solution $d^{\left(1\right)}$ using the max–min criterion:
  (a) For each solution $d_{i}$, determine the minimum of the standardized evaluations with respect to each criterion:
    (21) $$g^{\min}\left(d_{i}\right)=\underset{k\in \bar{1,K}}{\min}g^{k}\left(d_{i}\right)$$
  (b) As the first candidate solution $d^{\left(1\right)}$, take $d_{i}$ for which the value $g^{\min}\left(d_{i}\right)$ is maximal.
4. Set q=1, $D^{\left(1\right)}=D$, and $S=\left\{d^{\left(1\right)}\right\}$; determine the potency matrix $M^{\left(1\right)}$ and proceed to the first iteration.

*Iteration q*

1. Present the criteria values obtained for solution $d^{\left(q\right)}$ and potency matrix $M^{\left(q\right)}$ to the DM. If the DM is satisfied with the proposed solution, end the procedure.
2. Ask the DM if he/she wants to formulate additional requirements that the solution should meet. If the answer is positive, proceed to step 6.
3. Ask the DM if he/she would like to reconsider any of the previously proposed solutions. If the answer is negative, end the procedure.
4. Present to the DM the solutions proposed earlier and ask him/her to indicate which one he/she would like to reconsider.
5. As the next solution $d^{\left(q+1\right)}$, take the one indicated by the DM in step 4; set q=q+1 and proceed to the next iteration.
6. Ask the DM to assign each criterion $f^{k}$ to one of the following three sets:
  $F_{1}$—the set of criteria whose values should be improved as compared with the value obtained for solution $d^{\left(q\right)}$;
  $F_{2}$—the set of criteria whose values should not be made worse as compared with the value obtained for solution $d^{\left(q\right)}$;
  $F_{3}\;$—the set of criteria whose values can be made worse as compared with the value obtained for solution $d^{\left(q\right)}$.

We have:

(22) $$F=F_{1}\cup^{\;}F_{2}\cup^{\;}F_{3}$$

7. Determine the set $D^{\left(q+1\right)}$ as the set of solutions $d_{i}\in D^{\left(q\right)}$ satisfying the following conditions:
  (23) $$\begin{array}{c} \forall_{f^{k}\in F_{1}\cap^{\;}F_{\max}}\;f^{k}\left(d_{i}\right)>f^{k}\left(d^{\left(q\right)}\right),\\ \forall_{f^{k}\in F_{2}\cap^{\;}F_{\max}}\;f^{k}\left(d_{i}\right)\geq f^{k}\left(d^{\left(q\right)}\right),\\ \forall_{f^{k}\in F_{1}\cap^{\;}F_{\min}}\;f^{k}\left(d_{i}\right)<f^{k}\left(d^{\left(q\right)}\right),\\ \forall_{f^{k}\in F_{2}\cap^{\;}F_{\min}}\;f^{k}\left(d_{i}\right)\leq f^{k}\left(d^{\left(q\right)}\right). \end{array}$$
8. If $D^{\left(q+1\right)}=\emptyset$, notify the DM that there are no solutions satisfying his/her requirements, proceed to step 1.
9. Determine the potency matrix $M^{\left(q+1\right)}$ and ask the DM if he/she accepts the replacement of $M^{\left(q\right)}$ by $M^{\left(q+1\right)}$. If the answer is negative, proceed to step 1.
10. If $D^{\left(q+1\right)}$ consists of only one solution, take this solution as the next proposed solution $d^{\left(q+1\right)}$. Proceed to step 14.
11. For each solution $d_{i}\in D^{\left(q+1\right)}$ and for each criteria pair, such that $f^{k}\in F_{1},\;f^{l}\in F_{3}\;\mathrm{and}\;g^{l}\left(d_{i}\right)<g^{l}\left(d^{\left(q\right)}\right)$, calculate the value of the trade-off $t_{kl}\left(d_{i},d^{\left(q\right)}\right)$.
12. For each criteria pair $\left(f^{k},f^{l}\right)$ such that $f^{k}\in F_{1},\;f^{l}\in F_{3}$, check if there exists at least one solution $d_{i}$ for which the value of $t_{kl}\left(d_{i},d^{\left(q\right)}\right)$ has been calculated in step 11. If so, then for each solution $d_{j}\in D^{\left(q+1\right)}$ such that $g^{l}\left(d_{j}\right)\geq g^{l}\left(d^{\left(q\right)}\right)$, take as the trade-off $t_{kl}\left(d_{j},d^{\left(q\right)}\right)$ twice the maximal value of the trade-offs calculated for the pair $\left(f^{k},f^{l}\right)$ in step 11; otherwise, take $t_{kl}\left(d_{j},d^{\left(q\right)}\right)=1$ for each $d_{j}\in D^{\left(q+1\right)}$.
13. For each solution $d_{i}\in D^{\left(q+1\right)}$, calculate the average of the trade-offs calculated in steps 11 and 12 for each criteria pair $\left(f^{k},f^{l}\right)$ such that $f^{k}\in F_{1},\;f^{l}\in F_{3}$. As the next solution $d^{\left(q+1\right)}$ to be proposed to the DM, take the one for which this average is highest.
14. Set $S=S\cup^{\;}\left\{d^{\left(q+1\right)}\right\},\;\;q=q+1$ and proceed to the next iteration.

**Figure 1:**
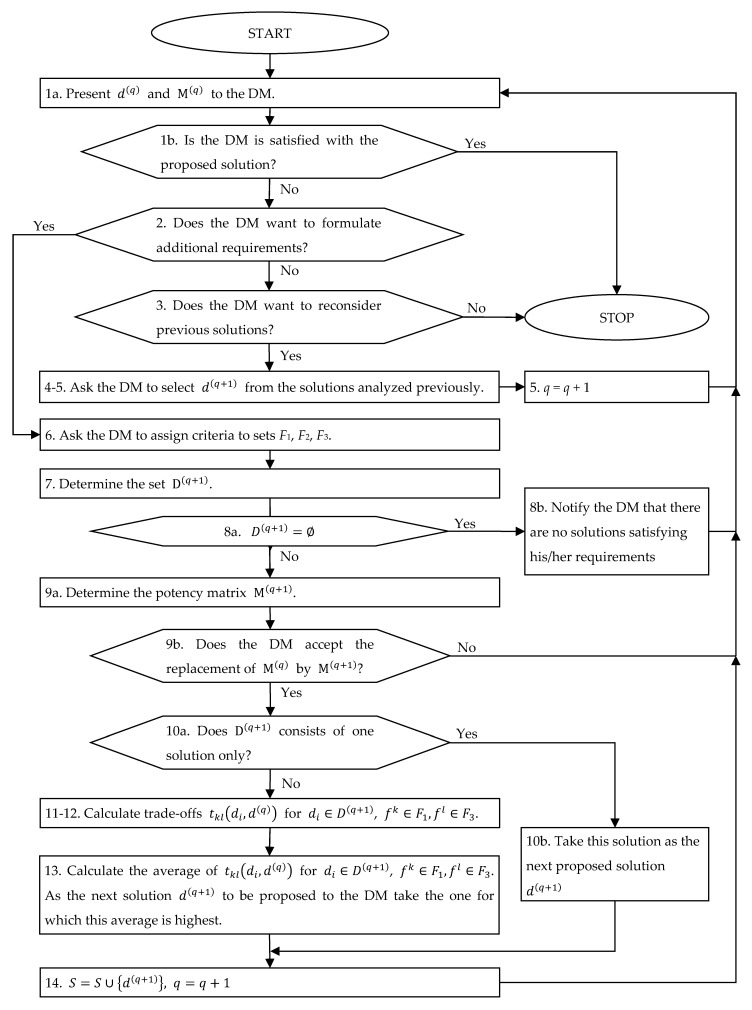
Flow chart of the iterative part of the procedure.

A flow chart of the iterative part of the procedure is presented in Figure 1.

### 3.3. Discussion

The first candidate solution is determined using the max–min criterion. In each iteration, the DM is presented with evaluations of the proposed solution and with the potency matrix consisting of maximal and minimal criteria values obtained for the currently considered solutions. The DM can either accept the proposed solutions as the solution of the problem or else determine the direction of improvement by indicating:

(a) The criteria which should achieve a value higher than the one obtained for the candidate solution;
(b) The criteria which should retain the value obtained for the candidate solution;
(c) The criteria which can have a lower value than the one obtained for the candidate solution.

Of course, since we operate within the set of efficient solutions, the DM must indicate at least one criterion whose value can be made worse.

The procedure should continue until the DM is satisfied with the proposed solution (step 1). During the dialog, it can turn out, however, that the consecutive proposals do not satisfy the DM’s expectations. He/she can then either end the procedure (step 3) or once again consider the solutions proposed earlier and decide to select one of them (step 4).

The procedure allows the DM to return to the solution which he/she previously regarded as unsatisfactory (steps 3 and 4). There is, of course, the danger that the DM will “fall into a loop” while searching for the solution. We assume, however, that the DM is aware of this danger. To abandon this possibility would exclude the risk of falling into a loop, but at the same time, it would substantially limit the flexibility of the procedure. It could also lead to the rejection of the determined solution by the DM as unsatisfactory. 

If in Step 3 the DM gave a negative answer, the procedure is halted without the final solution being determined. Such a situation occurs when the DM does not accept the currently proposed solution, and at the same time, does not intend to formulate additional requirements, nor does he/she want to return to any of the solutions proposed earlier.

In the procedure, it has been assumed that when the calculation of the trade-off for the given solution and the given criteria pair is not possible, we take for its value the double value of the maximal trade-off for the same criteria pair, but for different solutions. This is related to the fact that in such a case, it is possible to improve the value of criterion $f^{k}$ without making the value of criterion $f^{l}$ worse, which is, of course, very advantageous. It is also possible to take more than double the maximum, which allows for an even stronger preference of those solutions whose choice will not entail a decrease of the values of non-essential (for the DM) criteria.

## 4. Capacity Planning as a Mixed, Multiobjective Dynamic Programming Problem

Capacity planning is usually analyzed in a larger time horizon and can be considered as a discrete multistage decision process with a fixed number of stages. At the beginning of each stage, the process is in one of the feasible states, which is represented by the current production capacity. For each state, a set of feasible decisions is defined. Each decision determines the scale of the expansion of production capacities at the current stage. The state at the beginning of the next stage is determined by the transition function.

Let us denote:

*T*—Time horizon of the analysis (number of years);
*y_t_*—The production capacity at the beginning of stage *t*;
*x_t_* –Production capacity increment in stage *t.*

The transition function is as follows:

(24) $$y_{t+1}=y_{t}+x_{t}\;$$

Let us consider the following problem. A company introduces a new product to the market. The initial production capacity is 1000 units per year. It is forecasted that in the next five years the demand for the product will be increasing, and then it will level out at 5000 units. Therefore, the company prepares a long-term investment plan whose implementation will allow it to reach the full production capacity at the level of 5000 units no later than at the end of the fifth year. It is also assumed that after ten years, the production of the product will end. Because of the technology used, the value by which the production capacity is increased must be a multiple of 1000 units. Since the full production capacity has to be achieved at the end of the fifth year, we assume that the process considered has five stages, that is, *T* = 5.

Now we will determine the sets *Y_t_* of admissible states, for $t\in \bar{1,\;T}$, and of admissible decisions for the consecutive stages ***X****_t_*(*y_t_*). On the basis of our assumptions, we have:

Y_1_ = {1000}
*Y*_2_ = *Y*_3_ = *Y*_4_ = *Y*_5_ = {1000, 2000, 3000, 4000, 5000}
*Y*_6_ = {5000}

For stages 1 through 4, the sets of feasible decisions are:

*X_t_*(1000) = {0, 1000, 2000, 3000, 4000} *X_t_*(2000) = {0, 1000, 2000, 3000}
*X_t_*(3000) = {0, 1000, 2000}      *X_t_*(4000) = {0, 1000}
*X_t_*(5000) = {0}

In stage 5, the feasible decisions are:

*X*_5_(1000) = {4000} *X*_5_(2000) = {3000} *X*_5_(3000) = {2000}
*X*_5_(4000) = {1000} *X*_5_(5000) = {0}

A stage realization is a pair (*y*_t_, *x*_t_), where *x*_t_ ∈***X***_t_(*y_t_*). A process realization *d* is a sequence of stage realizations (*y*_1_, *x*_1_), (*y*_2_, *x*_2_), …, (*y*_T,_ *x*_T_), where *y*_t+1_ = *Ω*_t_(*y*_t_, *x*_t_) for *t*∈$\bar{1,\;T-1}$.

The evaluation of each process realization is an additive composition of stage evaluations for the individual stages.

When making decisions regarding the expansion of production capacity, many criteria are usually considered. Some of them are financial (for instance, Net Present Value or NPV), others allow evaluating the degree to which the company will be able to satisfy the demand in the future, the future demand for investment capital, or the future labor cost related to the investment projects being implemented.

We assume that the scope of the possible expansion of production capacity in the consecutive stages of the process analyzed has been determined on the basis of an analysis of technical and organizational constraints. In our model, five criteria are taken into account:

*α*^1^—Sum of discounted cash flow, to be maximized;
*α*^2^—Mean level of customer demand fulfillment in the analyzed period, to be maximized;
*α*^3^—Mean level of production capacity usage in the analyzed period, to be maximized;
*α*^4^—Total value of investment expenditure in the analyzed period, to be minimized;
*α*^5^—Total labor cost related to the preparation of the investment process, to be minimized.

We denote:

*z_t_*—Forecasted demand at stage *t*;
*s_t_*—Value of sales in stage *t*:

(25) $$s_{t}=\min\left\{y_{t},z_{t}\right\}$$

The value of the discounted cash flow in period *t* is calculated using the following formula [63]:

(26) $$\alpha_{t}^{1}\left(y_{t},x_{t}\right)=\frac{-I_{t}\left(x_{t}\right)+P_{t}\left(s_{t}\right)-K_{t}\left(y_{t},s_{t}\right)}{{\left(1+r\right)}^{t}}$$

where:

*I_t_*(*x_t_*)—Function describing the value of investment expenditure needed to increase the capacity by *x_t_* units;
*P_t_*(*s_t_*)—Function describing the value of sales revenue;
*K_t_*(*y_t_*, *s_t_*)—Function describing production costs;
*r*—discount rate. We assume that *k* = 0.1.

The values of criteria *α*^2^, *α*^3^, and *α*^4^ are determined as follows:

(27) $$\begin{array}{c} \alpha_{t}^{2}\left(y_{t},x_{t}\right)=\frac{s_{t}}{z_{t}}=\frac{min\left\{y_{t},z_{t}\right\}}{z_{t}}\\ \alpha_{t}^{3}\left(y_{t},x_{t}\right)=\frac{s_{t}}{y_{t}}=\frac{min\left\{y_{t},z_{t}\right\}}{y_{t}}\\ \alpha_{t}^{4}\left(y_{t},x_{t}\right)=I_{t}\left(x_{t}\right) \end{array}$$

The values of criterion *α*^5^ are determined by an expert.

On the basis of the data prepared by the marketing department, the demand forecasts for the product for the next five years are prepared (Table 2). 

A single capacity increase requires to cover the fixed cost of 1000 monetary units and, additionally, the variable cost of two monetary units for each unit of production capacity increase. The company sells the product at the price of five monetary units. Production cost depends on the production capacity installed and on the number of units produced. Each unit of the available production capacity entails the cost of one monetary unit, while the variable production cost is three per production unit. 

Because of fairly low employment, of essential importance is the labor expenditure related to the preparation of investment processes; its estimation is expressed by a fuzzy number. The data are presented in Table 3.

The value of investment expenditure is as follows:

(28) $$I_{t}\left(x_{t}\right)=1000\lambda \left(x_{t}\right)+2x_{t}$$

where:

(29) $$\lambda \left(x_{t}\right)=\{\begin{array}{cc} 1 & \mathrm{if}\;x_{t}>0\\ 0 & \mathrm{if}\;x_{t}=0 \end{array}$$

Revenue and production costs are determined as follows:

(30) $$\begin{array}{c} P_{t}\left(s_{t}\right)=5s_{t}\\ K_{t}\left(y_{t},s_{t}\right)=y_{t}+3s_{t} \end{array}$$

Since it is assumed that in the years 6 through 10 the demand will level out at 5000 units, the sum of discounted profits achieved in that period will be constant, regardless of the strategy of production capacity expansion adopted during the first five years. 

Criteria *α*^1^, *α*^2^, and *α*^3^ are stochastic: we assume that the company has a demand forecast for its products, which allows generating probability distributions for NPV, mean degree of demand fulfillment, and the mean level of production capacity usage. Criterion *α*^4^ is deterministic: we assume that the available data allow us to precisely determine the investments needed for the expansion of production capacity. Criterion *α*^5^ is fuzzy: the estimation of labor expenditure required to carry out the investment project is determined by an expert as a triangular fuzzy number. 

The operators are the same as in Example 1, described in Section 2. Moreover, the way of comparing random variables, real numbers, and triangular fuzzy numbers is the same as described there. The evaluation space is therefore a partially ordered space. Its structure corresponds to the structure presented in Example 1.

It is possible to find efficient realizations using the optimization principle and optimization equations derived for partially ordered spaces. These equations and proofs of the theorems can be found in [49,50]. A numerical solution of the problem has been presented in [64].

Efficient realizations *D* = {*d*_1_, …, *d*_25_}, determined by means of optimization equations, are presented in Table 4.

## 5. Application of the Interactive Procedure

The next three examples are illustrations of the procedure. Example 4 illustrates the preliminary stage, and example 5 illustrates three subsequent iterations.

The operators are the same as in Example 1, described in Section 2. Moreover, the way of comparing random variables, real numbers, and triangular fuzzy numbers is the same as described there. The evaluation space is, therefore, a partially ordered space. Its structure corresponds to the structure presented in Example 2. All efficient solutions are included in ***D***^(1)^.

**Example** **4.** *In the preliminary stage, calculations proceed as follows:*

*1.*  *Applying Formulas (25)–(30), for each efficient solution d_i_∈****D****^(1)^, we calculate*$f^{k}\left(d_{i}\right)$*,*$k\in \bar{1,\;K}$*,*$i\in \bar{1,\;N}$*. The results are given in*Table 5*in columns*$f^{1}\left(d_{i}\right)$*,*$f^{2}\left(d_{i}\right)$*,*$f^{3}\left(d_{i}\right)$*,*$f^{4}\left(d_{i}\right)$*,*$f^{5}\left(d_{i}\right)$.
*2.*  *Applying formula (17),for each efficient solution d_i_∈****D****^(1)^, we calculate*$g^{k}\left(d_{i}\right)$*,𝑘∈*$\bar{1,\;K}$*,*$i\in \bar{1,\;N}$*. The results are given in*Table 5*in columns*$g^{1}\left(d_{i}\right)$*,*$\;g^{2}\left(d_{i}\right)$*,*$\;g^{3}\left(d_{i}\right)$*,*$g^{4}\left(d_{i}\right)$*,*$\;g^{5}\left(d_{i}\right)$.
*3.*  *We determine the first candidate solution*$d^{\left(1\right)}$.
  (a) *For each solution *$d_{i}$*, we determine the minimum of the standardized evaluations with respect to each criterion, applying formula (21).*
  (b) *The value *$g^{min}\left(d_{i}\right)$*is maximal for i∈{12, 15, 16, 17, 18}. As the first candidate solution *$d^{\left(1\right)}$*, we take d_12_ (the first of the five solutions for which the minimum of the standardized criteria values is 0.667):*
    (31) $$d^{\left(1\right)}=d_{12}.$$
*4.*  *We set*q=1*,*$D^{\left(1\right)}=D$*,*$S=\left\{d^{\left(1\right)}\right\}$*, and determine the potency matrix*$M^{\left(1\right)}$*according to Formula (19). The potency matrix*$M^{\left(1\right)}$*is shown in*Table 6.

*Then, we proceed to the first iteration.*

**Example** **5.** *In iteration 1, the calculations proceed as follows*:

*1.*  *The criteria values for solution*$d^{\left(1\right)}$*and the potency matrix*$M^{\left(1\right)}$*(*Table 6*) are presented to the DM.Assume thatthe DM is not satisfied with solution*$d^{\left(1\right)}$.
*2.*  *The DM decides to formulate additional requirements—the procedure proceeds to step 6.*
*6.*  *The DM decides that the value of criterion f^1^ should be higher than 12911, while the values of f^2^ and f^3^ can be decreased, and the values of f^4^ and f^5^ should not be increased as compared with the ones obtained for solution* $d^{\left(1\right)}$ *:*
(32) $$F_{1}=\left\{f^{1}\;\right\},\;F_{2}=\left\{f^{4},f^{5}\right\},\;F_{3}=\left\{f^{2},f^{3}\right\}.$$
*7.*  *The set of solutions satisfying the conditions formulated by the DM in step 6 are determined:*
(33) $$D^{\left(2\right)}=\left\{d_{5},d_{14},d_{15},d_{16},d_{18}\right\}.$$
*8.*  *Since* $D^{\left(2\right)}\neq \emptyset$ *, we proceed to the next step.*
*9.*  *The potency matrix*$M^{\left(2\right)}$*is determined (*Table 7*); potency matrices*$M^{\left(1\right)}$*and*$M^{\left(2\right)}$*are presented to the DM, who accepts the move from*$M^{\left(1\right)}$*to*$M^{\left(2\right)}$.
*10.*  *Since*$D^{\left(2\right)}$*contains more than one solution, weproceed to the next step*.
*11.*  *Trade-offs* $t_{12}\left(d_{i},d^{\left(1\right)}\right)$ *and* $t_{13}\left(d_{i},d^{\left(1\right)}\right)$ *for* $d_{i}\in D^{\left(2\right)}\;are\;calculated$ *. When calculating the first value, we omit the solutions for which* $f^{2}\left(d_{i}\right)\geq f^{2}\left(d^{\left(1\right)}\right)$ *: d_5_ and d_14_. For the remaining solutions from* $D^{\left(2\right)}$ *the* *trade-offs* *are:*
(34) $$t_{12}\left(d_{15},d^{\left(1\right)}\right)=186.39,\;t_{12}\left(d_{16},d^{\left(1\right)}\right)=2.98,\;t_{12}\left(d_{18},d^{\left(1\right)}\right)=0.20.$$

*When calculating* $t_{13}\left(d_{i},d^{\left(1\right)}\right)$ *, we note that* $f^{3}\left(d_{i}\right)\geq f^{3}\left(d^{\left(1\right)}\right)\;$ *holds* *for d_16_ and d_18_* *. The* *trade-offs* *calculated for the other solutions are:*

(35) $$t_{13}\left(d_{5},d^{\left(1\right)}\right)=0.43,\;t_{13}\left(d_{14},d^{\left(1\right)}\right)=0.83,\;t_{13}\left(d_{15},d^{\left(1\right)}\right)=4.72.$$

*12.*  *For*$d_{5}$*and*$d_{14}$*, for whichtrade-offs* $t_{12}\left(d_{i},d^{\left(1\right)}\right)$*have not been calculated in step 7, we take:*
  (36) $$t_{12}\left(d_{5},d^{\left(1\right)}\right)=t_{12}\left(d_{14},d^{\left(1\right)}\right)=372.78$$

*Using the same rule, we calculate trade-offs* $t_{13}\left(d_{i},d^{\left(1\right)}\right)$ *for d_16_ and d_18_:*

(37) $$t_{13}\left(d_{16},d^{\left(1\right)}\right)=t_{13}\left(d_{18},d^{\left(1\right)}\right)=9.44.$$

*13.*  *The average values of the* *trade-offs* *are calculated (* Table 8 *).*

*The next solution d^(2)^ proposed to the DM is*$d_{14}$.

*14.*  *We set *$S=\left\{d_{12},d_{14}\right\}$*, *q=2* and proceed to the second iteration.*

*In iteration 2, the calculations proceed as follows:*

*1.*  *The criteria values for solution d^(2)^ and potency matrix* $M^{\left(2\right)}$ *(* Table 9 *) are presented to the DM.*

*The DM is not satisfied with solution d^(2)^.*

*2.*  *The DM decides to formulate additional requirements—the procedure proceeds to step 6.*
*6.*  *The DM decides that the value of criterion f ^3^ should be higher than 87.81%* *, while the values of the remaining criteria can be made worse (smaller for f^1^ and f^2^ and larger for f^4^ and f^5^) with respect to the values admitted by these criteria for d^(2)^:*
(38) $$F_{1}=\left\{f^{3}\right\},\;F_{2}=\emptyset ,\;F_{3}=\left\{f^{1},f^{2},f^{4},f^{5}\right\}.$$
*7.*  *The set of solutions satisfying the conditions formulated by the DM in step 3 are determined:*
(39) $$D^{\left(3\right)}=\left\{d_{15},d_{16},d_{18}\right\}.$$
*8.*  *Since* $D^{\left(3\right)}\neq \emptyset$ *, we proceed to the next step.*
*9.*  *The potency matrix*$M^{\left(3\right)}$*is determined (*Table 10*); potency matrices*$M^{\left(2\right)}$*and*$M^{\left(3\right)}$*are presented to the DM, who accepts the move from*$M^{\left(2\right)}$*to*$M^{\left(3\right)}$.
*10.*  *Since*$D^{\left(3\right)}$*contains more than one solution, weproceed to the next step*.
*11.*  *Trade-offs* $t_{31}\left(d_{i},d^{\left(2\right)}\right)$ *,* $t_{32}\left(d_{i},d^{\left(2\right)}\right)$ *,* $t_{34}\left(d_{i},d^{\left(2\right)}\right)$ *, and* $t_{35}\left(d_{i},d^{\left(2\right)}\right)$ *for* $d_{i}\in D^{\left(3\right)}$ *are calculated. The values of f ^5^ for d_18_ is the same as for d^(2)^, which means that* $t_{35}\left(d_{18},d^{\left(2\right)}\right)$ *cannot be calculated.*
*12.*  *As* $t_{35}\left(d_{18},d^{\left(2\right)}\right)$ *, we take twice the maximum value of the values of trade-offs calculated for the pair (f ^3^, f ^5^) in step 7.*
*13.*  *The average values of the* *trade-offs* *are calculated (* Table 11 *).*

*The next solution d^(3)^ proposed to the DM is*$d_{16}$.

*14.*  *We set *$S=\left\{d_{12},d_{14},d_{16}\right\}$*,*q=3* and proceed to the next iteration.*

*In iteration 3, the calculations proceed as follows:*

*1.*  *The criteria values for solution d^(3)^ and the potency matrix* $M^{\left(3\right)}$ *(* Table 12 *) are presented to the DM.*

*The DM is satisfied with solution d^(3)^. The procedure ends.*

The solution determined means that the production capacity should be increased by 3000 units in the second year and by 1000 units in the fourth year.

## 6. Conclusions

To determine the final solution to the problem, we use an interactive procedure. Its basic advantage consists of not being very demanding on the DM, who, after having obtained the solution proposed, should determine whether it is satisfactory, and if not, how it should be improved. It is expected that the DM will indicate the criteria whose values should be improved, the criteria whose values cannot be worsened, and the criteria whose values can be worse than the ones obtained for the solution considered.

The interactive method proposed in the paper is an improved version of the procedure presented by the present authors in their earlier paper [65]. The changes, as compared with the previous versions, are as follows. First, in the current version, both the criteria to be maximized and those to be minimized are taken into account (in the earlier versions, we focused only on the former). Second, in the current version, the decision-maker can compare the current potency matrix with the one resulting from taking into account the requirements formulated by him/her and from accepting (or not) the direction of the improvements of the solution obtained in the current iteration. Third, all the solutions proposed to the DM are saved so that it is possible to return easily to any of the previous ones—should the DM decide that an earlier solution better satisfies his/her expectations than a solution proposed later—and to propose this solution as the final one. 

In this paper, we also present how the procedure can be applied to capacity planning, which is of crucial importance for achieving the strategic goals of an organization. When making decisions regarding the expansion of the production capacity, managers usually take into account many criteria of diverse character. Depending on the method of obtaining data, criteria can be deterministic, stochastic, or fuzzy. In this paper, we have shown that this problem can be formulated as a mixed multiobjective dynamic programming problem. We have also proposed a method that can be used to solve this problem.

A certain flaw of our method is its fairly simple way of scalarization of the stochastic and fuzzy criteria. In further papers, we plan to propose new methods of interacting with the DM, which will allow us to present more extensive information on the expected effects of such criteria. In particular, in the case of stochastic criteria, we intend to provide the DM not only with the expected value but also with the values of the individual quantiles of the distribution of evaluation.

## Figures and Tables

**Table 1:** Example solutions of the problem.

Solution	*f*^1^ (max)	*f*^2^ (max)	*f*^3^ (max)
*d*_1_	10	20	15
*d*_2_	12	14	16
*d*_3_	14	22	8
*d*_4_	18	12	10

**Table 2:** Demand for the product.

Year 1		Year 2		Year 3		Year 4		Year 5	
Demand	P(*z_t_*)	Demand	P(*z_t_*)	Demand	P(*z_t_*)	Demand	P(*z_t_*)	Demand	P(*z_t_*)
650	0.15	1880	0.20	3470	0.25	3930	0.30	4320	0.35
710	0.80	2130	0.65	4090	0.55	4770	0.45	4990	0.35
790	0.05	2510	0.15	4880	0.20	5000	0.25	5000	0.30

**Table 3:** Estimation of the labor expenditure related to the preparation of the investment process: the number of man-hours (*m* is the center of the fuzzy number, *α*, *β* are spreads).

Production Capacity Increment	Fuzzy Number Parameters		
	*α*	*M*	*β*
0	0	0	0
1000	20	200	80
2000	40	240	100
3000	60	320	130
4000	90	480	140

**Table 4:** Efficient process realizations.

Process Realization	*y*_1_	*x*_1_	*y*_2_	*x*_2_	*y*_3_	*x*_3_	*y*_4_	*x*_4_	*y*_5_	*x*_5_	*y*_6_
*d*_1_	1000	4000	5000	0	5000	0	5000	0	5000	0	5000
*d*_2_	1000	2000	3000	2000	5000	0	5000	0	5000	0	5000
*d*_3_	1000	2000	3000	1000	4000	1000	5000	0	5000	0	5000
*d*_4_	1000	2000	3000	0	3000	2000	5000	0	5000	0	5000
*d*_5_	1000	1000	2000	3000	5000	0	5000	0	5000	0	5000
*d*_6_	1000	1000	2000	2000	4000	1000	5000	0	5000	0	5000
*d*_7_	1000	1000	2000	2000	4000	0	4000	1000	5000	0	5000
*d*_8_	1000	1000	2000	2000	4000	0	4000	0	4000	1000	5000
*d*_9_	1000	1000	2000	1000	3000	1000	4000	0	4000	1000	5000
*d*_10_	1000	1000	2000	1000	3000	0	3000	1000	4000	1000	5000
*d*_11_	1000	1000	2000	1000	3000	0	3000	0	3000	2000	5000
*d*_12_	1000	1000	2000	0	2000	3000	5000	0	5000	0	5000
*d*_13_	1000	1000	2000	0	2000	2000	4000	0	4000	1000	5000
*d*_14_	1000	0	1000	4000	5000	0	5000	0	5000	0	5000
*d*_15_	1000	0	1000	3000	4000	1000	5000	0	5000	0	5000
*d*_16_	1000	0	1000	3000	4000	0	4000	1000	5000	0	5000
*d*_17_	1000	0	1000	3000	4000	0	4000	0	4000	1000	5000
*d*_18_	1000	0	1000	2000	3000	2000	5000	0	5000	0	5000
*d*_19_	1000	0	1000	2000	3000	1000	4000	0	4000	1000	5000
*d*_20_	1000	0	1000	2000	3000	0	3000	2000	5000	0	5000
*d*_21_	1000	0	1000	2000	3000	0	3000	1000	4000	1000	5000
*d*_22_	1000	0	1000	2000	3000	0	3000	0	3000	2000	5000
*d*_23_	1000	0	1000	0	1000	4000	5000	0	5000	0	5000
*d*_24_	1000	0	1000	0	1000	0	1000	4000	5000	0	5000
*d*_25_	1000	0	1000	0	1000	0	1000	0	1000	4000	5000

**Table 5:** Scalarized values of criteria for efficient realizations and standardized values.

Solution	*f*^1^(*d_i_*)	*f*^2^(*d_i_*)	*f*^3^(*d_i_*)	*f*^4^(*d_i_*)	*f*^5^(*d_i_*)	*g*^1^(*d_i_*)	*g*^2^(*d_i_*)	*g*^3^(*d_i_*)	*g*^4^(*d_i_*)	*g*^5^(*d_i_*)	Min
*d*_1_	11,393	100.00%	76.36%	9000	480	0.474	1.000	0.000	1.000	1.000	0.000
*d*_2_	12,550	100.00%	82.05%	10,000	480	0.755	1.000	0.321	0.667	1.000	0.321
*d*_3_	12,361	99.04%	85.02%	11,000	640	0.709	0.983	0.488	0.333	0.500	0.333
*d*_4_	12,786	94.85%	85.68%	10,000	480	0.812	0.910	0.526	0.667	1.000	0.526
*d*_5_	13,276	98.60%	87.57%	10,000	520	0.930	0.975	0.632	0.667	0.875	0.632
*d*_6_	13,087	97.63%	90.53%	11,000	640	0.885	0.958	0.799	0.333	0.500	0.333
*d*_7_	13,161	95.18%	92.13%	11,000	640	0.902	0.915	0.889	0.333	0.500	0.333
*d*_8_	13,026	92.07%	93.09%	11,000	640	0.870	0.861	0.943	0.333	0.500	0.333
*d*_9_	11,873	87.89%	93.76%	12,000	800	0.590	0.787	0.981	0.000	0.000	0.000
*d*_10_	11,423	83.58%	93.86%	12,000	800	0.482	0.712	0.986	0.000	0.000	0.000
*d*_11_	11,609	79.36%	93.86%	11,000	640	0.527	0.638	0.986	0.333	0.500	0.333
*d*_12_	12,911	88.50%	91.20%	10,000	520	0.842	0.798	0.836	0.667	0.875	0.667
*d*_13_	12,098	82.94%	93.76%	11,000	640	0.645	0.701	0.981	0.333	0.500	0.333
*d*_14_	13,564	89.43%	87.81%	9000	480	1.000	0.814	0.645	1.000	1.000	0.645
*d*_15_	13,375	88.46%	90.77%	10,000	520	0.954	0.798	0.813	0.667	0.875	0.667
*d*_16_	13,448	86.01%	92.37%	10,000	520	0.972	0.755	0.902	0.667	0.875	0.667
*d*_17_	13,313	82.90%	93.33%	10,000	520	0.939	0.700	0.957	0.667	0.875	0.667
*d*_18_	12,973	84.28%	91.44%	10,000	480	0.857	0.724	0.850	0.667	1.000	0.667
*d*_19_	12,160	78.72%	94.00%	11,000	640	0.660	0.627	0.994	0.333	0.500	0.333
*d*_20_	12,528	77.52%	93.13%	10,000	480	0.749	0.605	0.946	0.667	1.000	0.605
*d*_21_	11,711	74.41%	94.10%	11,000	640	0.551	0.551	1.000	0.333	0.500	0.333
*d*_22_	11,897	70.19%	94.10%	10,000	480	0.596	0.477	1.000	0.667	1.000	0.477
*d*_23_	12,597	74.38%	91.44%	9000	620	0.766	0.550	0.850	1.000	1.000	0.550
*d*_24_	11,060	58.79%	93.13%	9000	620	0.394	0.277	0.946	1.000	1.000	0.277
*d*_25_	9435	43.01%	94.10%	9000	620	0.000	0.000	1.000	1.000	1.000	0.000

**Table 6:** Candidate solution *d*^(1)^ and potency matrix M^(1)^.

	*f*^1^ (max)	*f*^2^ (max)	*f*^3^ (max)	*f*^4^ (min)	*f*^5^ (min)
*d* ^(1)^	12,911	88.50%	91.20%	10,000	520
${\bar{f}}^{k\left(1\right)}$	13,564	100.00%	94.10%	9000	480
${\underline{f}}^{k\left(1\right)}$	9435	43.01%	76.36%	12,000	800

**Table 7:** Potency matrix M^(2)^.

	*f*^1^ (max)	*f*^2^ (max)	*f*^3^ (max)	*f*^4^ (min)	*f*^5^ (min)
${\bar{f}}^{k\left(2\right)}$	13,564	98.60%	92.37%	9000	480
${\underline{f}}^{k\left(2\right)}$	12,973	84.28%	87.57%	10,000	520

**Table 8:** Values of the trade-offs in iteration 1.

Solution	*t*_12_(*d_i_*, *d*^(1)^)	*t*_13_(*d_i_*, *d*^(1)^)	Average
*d*_5_	372.78	0.43	186.61
*d*_14_	372.78	0.83	186.81
*d*_15_	186.39	4.72	95.56
*d*_16_	2.98	9.44	6.21
*d*_18_	0.20	9.44	4.82

**Table 9:** Candidate solution *d*^(2)^ and potency matrix M^(2)^.

	*f*^1^ (max)	*f*^2^ (max)	*f*^3^ (max)	*f*^4^ (min)	*f*^5^ (min)
*d* ^(2)^	13,564	89.43%	87.81%	9000	480
${\bar{f}}^{k\left(2\right)}$	13,564	98.60%	92.37%	9000	480
${\underline{f}}^{k\left(2\right)}$	12,973	84.28%	87.57%	10,000	520

**Table 10:** Potency matrix M^(3)^.

	*f*^1^ (max)	*f*^2^ (max)	*f*^3^ (max)	*f*^4^ (min)	*f*^5^ (min)
${\bar{f}}^{p\left(3\right)}$	13,448	88.46%	92.37%	10,000	520
${\underline{f}}^{p\left(3\right)}$	12,973	84.28%	90.77%	10,000	480

**Table 11:** Values of the trade-offs in iteration 2.

Solution	*t*_31_(*d_i_*, *d*^(2)^)	*t*_32_(*d_i_*, *d*^(2)^)	*t*_34_(*d_i_*, *d*^(2)^)	*t*_35_(*d_i_*, *d*^(2)^)	Average
*d*_15_	3.66	9.89	0.50	1.34	3.85
*d*_16_	9.18	4.29	0.77	2.06	4.08
*d*_18_	1.43	2.26	0.61	4.11	2.10

**Table 12:** Candidate solution *d*^(3)^ and potency matrix M^(3)^.

	*f*^1^ (max)	*f*^2^ (max)	*f*^3^ (max)	*f*^4^ (min)	*f*^5^ (min)
*d* ^(3)^	13,448	86.01%	92.37%	10,000	520
${\bar{f}}^{k\left(3\right)}$	13,448	88.46%	92.37%	10,000	520
${\underline{f}}^{k\left(3\right)}$	12,973	84.28%	90.77%	10,000	480

## Data Availability

Not applicable.
